# Dual antiplatelet therapy with clopidogrel and aspirin increases mortality in 4T1 metastatic breast cancer-bearing mice by inducing vascular mimicry in primary tumour

**DOI:** 10.18632/oncotarget.24891

**Published:** 2018-04-03

**Authors:** Marta Smeda, Anna Kieronska, Bartosz Proniewski, Agnieszka Jasztal, Anna Selmi, Krystyna Wandzel, Agnieszka Zakrzewska, Tomasz Wojcik, Kamil Przyborowski, Katarzyna Derszniak, Marta Stojak, Dawid Kaczor, Elzbieta Buczek, Cezary Watala, Joanna Wietrzyk, Stefan Chlopicki

**Affiliations:** ^1^ Jagiellonian Centre for Experimental Therapeutics, Jagiellonian University, Bobrzynskiego 14, Krakow 30-348, Poland; ^2^ Department of Haemostasis and Haemostatic Disorders, Medical University of Lodz, Kosciuszki 4, Lodz 90-419, Poland; ^3^ Hirszfeld Institute of Immunology and Experimental Therapy, Polish Academy of Sciences, Department of Experimental Oncology, Rudolfa Weigla 4, Wroclaw 53-114, Poland; ^4^ Chair of Pharmacology, Jagiellonian University, Medical College, Grzegorzecka 16, Krakow 31-531, Poland

**Keywords:** mouse breast cancer, vascular mimicry, platelet inhibition, aspirin, clopidogrel

## Abstract

Platelet inhibition has been considered an effective strategy for combating cancer metastasis and compromising disease malignancy although recent clinical data provided evidence that long-term platelet inhibition might increase incidence of cancer deaths in initially cancer-free patients. In the present study we demonstrated that dual anti-platelet therapy based on aspirin and clopidogrel (ASA+Cl), a routine regiment in cardiovascular patients, when given to cancer-bearing mice injected orthotopically with 4T1 breast cancer cells, promoted progression of the disease and reduced mice survival in association with induction of vascular mimicry (VM) in primary tumour. In contrast, treatment with ASA+Cl or platelet depletion did reduce pulmonary metastasis in mice, if 4T1 cells were injected intravenously. In conclusion, distinct platelet-dependent mechanisms inhibited by ASA+Cl treatment promoted cancer malignancy and VM in the presence of primary tumour and afforded protection against pulmonary metastasis in the absence of primary tumour. In view of our data, long-term inhibition of platelet function by dual anti-platelet therapy (ASA+Cl) might pose a hazard when applied to a patient with undiagnosed and untreated malignant cancer prone to undergo VM.

## INTRODUCTION

Platelets contribute to tumour cell growth as well as metastatic spread [1]. An increase in platelet number inversely correlates with cancer patient survival [2]. Furthermore, defective platelet function or reduced platelet count is associated with decreased metastasis [3–5]. Given the possible role of platelets in promotion of malignant diseases and metastatic spread, effects of anti-platelet therapy on metastasis have been intensively studied in animal models [3, 6, 7] as well as in humans. Today there is overwhelming evidence from epidemiological studies [8–12] and clinical trials [13, 14] that supports the involvement of platelets in metastatic spread.

Aspirin, inhibiting cyclooxygenase-1 (COX-1) –derived thromboxane A_2_ (TXA_2_) synthesis in platelets (and thus platelet aggregation and degranulation) was the first anti-platelet drug used to combat cancer metastasis [15]. There are a number of reports in experimentally introduced tumours and in clinical trials that demonstrate cancer-preventive [16–19] and anti-metastatic effects of aspirin [10, 20], although the latter effect was not consistent in all studies [2]. In cardiovascular patients, aspirin is frequently administered together with thienopyridines (for example clopidogrel) that antagonize P2Y_12_ receptors and prevent ADP-induced platelet activation. Surprisingly, recent clinical data suggest that long-term treatment with clopidogrel and other thienopyridines could increase the number of non-cardiovascular deaths, half of them were attributed to cancer [9, 14]. Moreover, inhibition of thrombin generation by vorapaxar applied on top of conventional anti-platelet therapy based on combination of aspirin and clopidogrel was also associated with increased cancer incidence in initially cancer-free cardiovascular patients [21]. Finally, it has been recently reported that some anticoagulants (warfarin) [22] or, surprisingly, platelet depletion [23] could increase metastatic burden in mice. Accordingly, in contrast with the numerous pre-clinical studies that have repeatedly shown slower progression of malignant diseases upon treatment with anti-platelet agents, recent evidence suggests that platelet inhibition with clopidogrel or vorapaxar might accelerate cancer progression in patients. In fact, the emerging controversy regarding the beneficial vs detrimental effects of platelet inhibition on progression of cancer has recently been documented in clinical trials [9, 14, 21]. Hence, we tested a hypothesis that platelet inhibition with aspirin and clopidogrel has differential effects on the primary tumour, as opposed to metastatic spread. We investigated the effects of dual anti-platelet therapy based on aspirin and clopidogrel on primary tumour growth in the murine model of 4T1 breast cancer after orthotopic injection of 4T1 breast cancer cells in comparison to the effects of aspirin and clopidogrel treatment on pulmonary metastasis after intravenous injection of 4T1 breast cancer cells. Our results clearly demonstrate differential effects of platelet inhibition with aspirin and clopidogrel on primary breast cancer growth and pulmonary metastasis. We demonstrated that platelet-dependent mechanisms inhibited by dual anti-platelet treatment with aspirin and clopidogrel promoted cancer malignancy and VM in the presence of primary tumour and afforded protection against pulmonary metastasis in the absence of primary tumour.

## RESULTS

### Effects of aspirin and clopidogrel on mice survival in the orthotopic model of metastatic 4T1 breast cancer

**Figure 1:**
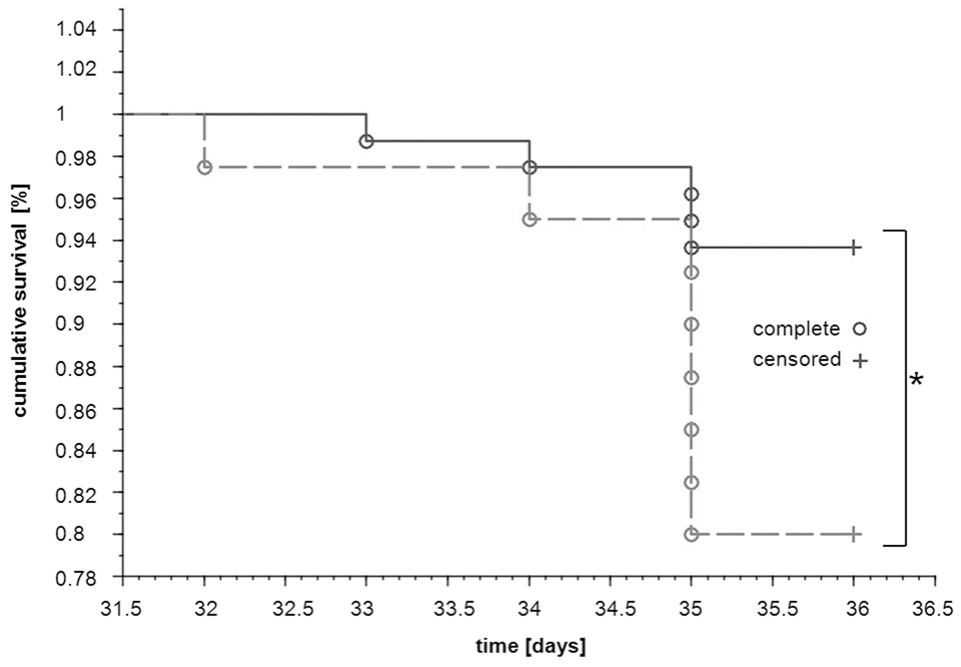
Effects of dual anti-platelet therapy with aspirin and clopidogrel on mice survival after orthotopic injection of 4T1 cells Mice were injected orthotopically with 1x10^4^ of 4T1 cells (n=120). Once the primary tumour was palpable, the designated group of animals started to receive aspirin (ASA) and clopidogrel (Cl) with the chow diet (12 mg +12 mg per kg of body weight per day) (dotted line; 4T1+ASA/Cl mice, n=40) whereas others continued on the standard chow (solid line; 4T1 mice, n=80). The disease progression was monitored throughout the experiment, and the number of days survived was registered for each animal until both groups were euthanized terminally in the 5^th^ week of the disease. Survival curves of both groups were compared with the generalised Wilcoxon (Peto-Prentice) test. The symbol ^*^ indicates statistical significance at P<0.05.

Survival of tumour-bearing mice receiving dual anti-platelet therapy based on aspirin (ASA) and clopidogrel (Cl) (4T1+ASA/Cl group, n=40) was diminished as compared with control tumour-bearing mice not subjected to the ASA+Cl treatment (4T1 control group, n=80) (P=0.0259) (Figure 1). Concomitantly, in ASA+Cl-treated mice, lung expression of negative prognostic factors of malignancy such as transforming growth factor β1 (TGFβ1), cyclooxygenase-2 (COX-2) and prostacyclin synthase (PGI_2_S) (41-43), was higher, despite the lower level of tissue remodeling/angiogenesis markers such as metaloproteinases/vascular endothelial growth factor A (VEGFA) (Supplementary Figure 1). Interestingly, there was no difference in body mass, primary tumour weight, primary tumour volume, lung weight, number of pulmonary metastases, relative pulmonary metastatic area and WBC count between 4T1+ASA/Cl and 4T1 control mice (Table 1). However, the number of circulating platelets was higher in 4T1+ASA/Cl mice, and correlated with a higher number of megakaryocytes in the spleen. Systemic NO bioavailability was also higher in 4T1+ASA/Cl mice, as evidenced by higher plasma NO_2_^-^, NO_3_^-^concentrations and higher HbNO levels in blood (Table 1).

**Table 1:** Effects of dual anti-platelet therapy with aspirin and clopidogrel on cancer progression, blood cell count and NO bioavailability in orthotopic metastatic breast cancer model

Parameter	Experimental groups	
	4T1	4T1+ASA/Cl
Body mass-primary tumour [g]	19.6±1.8 (n=53)	18.9±1.7 (n=17)
Primary tumour weight [g]	2.00±1.03 (n=68)	2.07±0.08 (n=26)
Primary tumour volume [mm^3^]	1205.0±419.4 (n=60)	1145.0±455.0 (n=40)
Lung weight [% body weight]	1.9±0.8 (n=49)	2.0±0.9 (n=26)
Number of metastases on the lungs’ surface	60; 55.0-75 (n=14)	62; 36-82 (n=9)
Number of metastases on H&E-stained lung cross-sections	59; 51-90 (n=10)	84; 38-109 (n=8)
Area of pulmonary metastases [% of lung cross-section area]	29.3±9.6% (n=10)	20.7±10.9% (n=8)
WBC [K · μl^-1^]	249.7±151.0 (n=36)	293.8±102.2 (n=8)
GRA [K · μl^-1^]	152.4±95.2 (n=34)	182.7±54.8 (n=8)
LYM [K · μl^-1^]	63.4±51.3 (n=35)	71.9±29.8 (n=8)
PLT [K · μl^-1^]	907.5±191.3 (n=56)	1026.0±161.3 (n=16)^*^ (P=0.022)
Number of megakaryocytes per eyefield in the spleen	12.7; 11.0-14.5 (n=13)	15.4; 13.0-17.0 (n=9)^*^ (P=0.048)
HbNO [AU per mg]	371.4±157.7 (n=23)	532.0±199.7 (n= 9) (P=0.024)^*^
NO_2_^-^ [μM]	0.36±0.1 (n=22)	0.84±0.50 (n=10) (P=0.003)^**^
NO_3_^-^ [μM]	14.7±5.7 (n=23)	20.1±6.2 (n=10) (P=0.022)^*^

Data are presented as mean ± SD or median and IQR. Body mass and primary tumour volume of mice injected with 1×10^4^ 4T1 cells (4T1) and those injected with 4T1 cells and receiving dual anti-platelet therapy (4T1+ASA/Cl) were monitored throughout the experiment. Mice were euthanized in the 5^th^ week of the disease, the blood cell count was performed and the lungs were excised for further analysis of pulmonary metastasis; primary tumours were excised and weighed. Pulmonary metastasis was assessed by counting the number of secondary nodules on formalin-fixed lung lobe surfaces under a magnifying glass. Subsequently, the lobes were paraffin-embedded, cut into slices and stained with H&E. The slices were scanned with a BX51 microscope equipped with virtual microscopy system dotSlide (Olympus, Japan), and the number of pulmonary metastases was counted concomitantly with the total area of pulmonary metastasis expressed as the percentage of lung cross-section area. Spleens were excised, weighed and fixed in formaldehyde, paraffin-embedded, cut into slices and stained with H&E. The slices were photographed and the number of megakariocytes was counted, as described in *Materials and Methods*. Systemic NO bioavailability was assessed by measurement of HbNO adducts in RBC as well as plasma NO_2_^-^ and NO_3_^-^ concentrations. The data were analyzed with two-sided Student *T* test or the non-parametric Mann-Whitney *U* test depending on the variable scale, the normality of distribution and the variance homogeneity (*F* test). The symbols ^*^ and ^**^ indicate statistical significance at, respectively, P<0.05 and P<0.01.

### Effects of aspirin and clopidogrel on pro-thrombotic phenotype of platelets in the orthotopic model of metastatic 4T1 breast cancer

**Figure 2:**
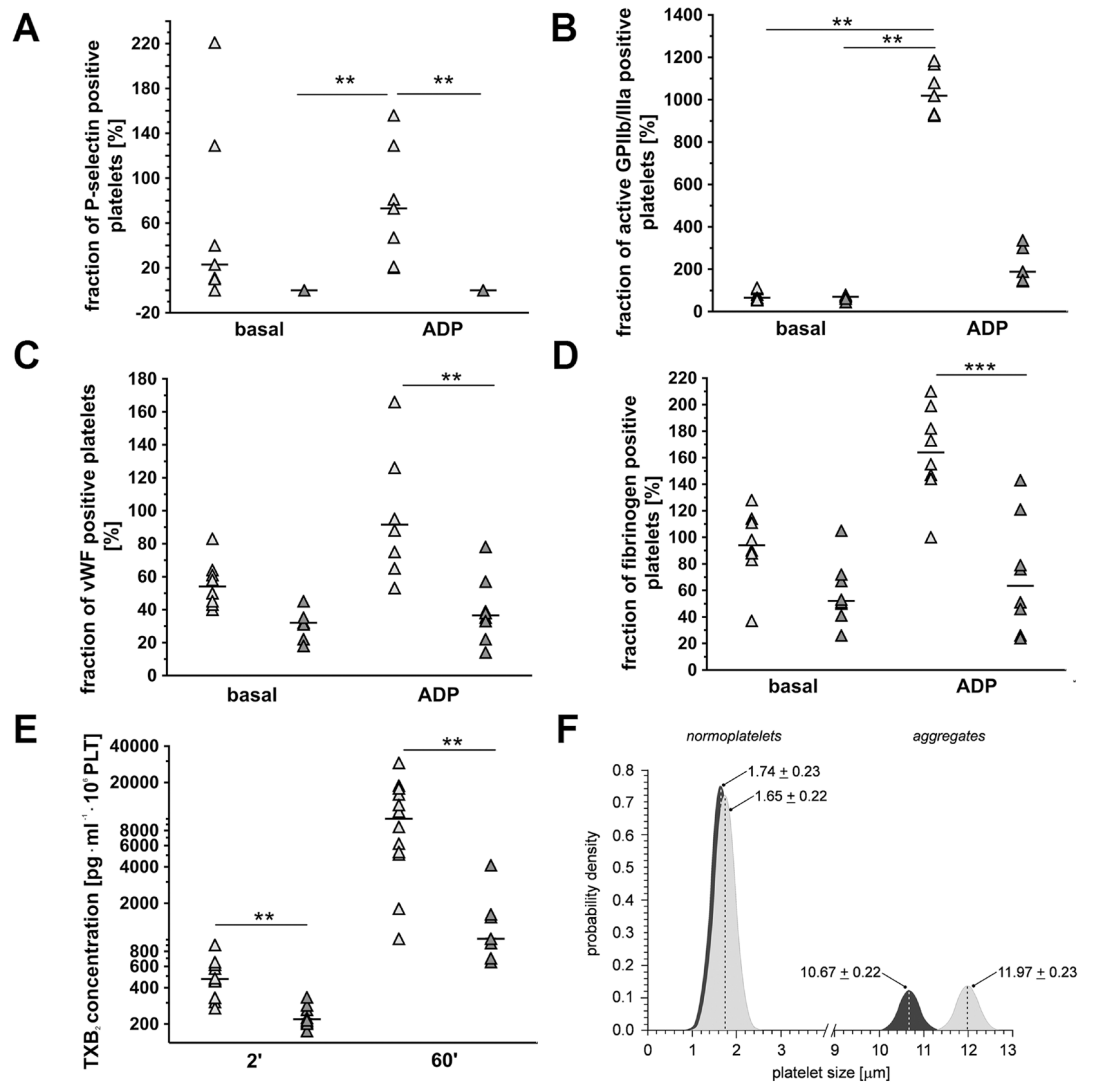
Effects of dual anti-platelet therapy with aspirin and clopidogrel on pro-thrombotic phenotype of platelets in orthotopic metastatic breast cancer model In the case of **(A-D)**, results are presented as median of data without outliers; n=7-8 for control 4T1 mice (light grey), n=5-8 for ASA+Cl-treated 4T1 mice (dark grey), in the case of **(E)** results are presented as median of data without outliers; n=12 for control 4T1 mice and n=7-8 for ASA+Cl-treated 4T1 mice, in the case of **(F)** as mean + SD or median and IQR; n=7 for control 4T1 mice and n = 7 for ASA+Cl-treated 4T1 mice. Mice were randomly assigned to control not-treated group (4T1; light grey) or the group that received anti-platelet therapy (4T1+ASA-Cl; dark grey). Platelet activation and their ADP-induced reactivity were assessed by exposure of their surface antigens P-selectin (A), active form of the receptor GPIIb/IIIa (B), von Willebrandt factor (vWF) binding, (C) and fibrinogen binding (D) to visualize the effectiveness of clopidogrel treatment. Based on the normality of distribution and variance homogeneity (Barlett’s test), the medians in (A-C) were compared with Kruskal-Wallis test followed by Dunn’s multiple comparison test; data shown in (D) were analysed with Two-Way ANOVA followed by Bonferroni *post-hoc* multiple comparisons test, the data of TXB_2_ (E) were analysed with Mann-Whitney *U* test to visualize effectiveness of aspirin treatment. (F) The sizes of CD61/CD41-positive objects in subpopulations of normoplatelets (cluster 1) and aggregates (cluster 2) in control (light grey) and ASA+Cl-treated (dark grey) 4T1 mice were assessed to confirm that platelet inhibition was associated with decreased formation of aggregates in the circulation of 4T1+ASA/Cl mice. Objects were discriminated based on flow cytometry forward scatter data, with the use of the Data Mining-assisted generalized cluster analysis by EM (expectation-maximization) algorithm decomposing the overall population into two exponentially-modified Gaussian partial distributions relevant to platelet subpopulations representing small objects (normoplatelets, predominantly single blood platelets, subpopulation 1) and larger objects (platelet aggregates with other blood cells, subpopulation 2) in 4T1 and ASA+CL-treated 4T1 mice. The contributions of normoplatelets and aggregates were 83.8% and 16.2% in 4T1 controls *vs*. 86.0% *vs*. 14.0% in 4T1 mice treated with ASA+CL (P< 0.0001 for aggregates: 16.2; 15.1-18.3% in control *vs*. 14.0; 13.3-14.8% in 4T1+ASA/Cl mice, by the bootstrap-boosted estimate of the one-sided Mann-Whitney *U* test, 1000 iterations). The symbols ^**^ and ^***^ indicate statistical significance at, respectively, P<0.01 and P<0.001.

To confirm effectiveness of dual anti-platelet ASA+Cl therapy, basal and ADP-induced platelet reactivity (Figure 2A-2D), *ex vivo* thromboxane B_2_ (TXB_2_) generation (Figure 2E) and platelet aggregates formation (Figure 2F) were measured. As shown in Figure 2A-2D, anti-platelet therapy in 4T1+ASA/Cl group attenuated ADP-induced increase in platelet P-selectin (n=7-5, P<0.05), vWF (n=8, P<0.001) and fibrinogen binding (n=8, P<0.001) on the platelet surface when compared with ADP-stimulated platelets in 4T1 control mice. Anti-platelet treatment inhibited also *ex vivo* TXB_2_ generation in 4T1+ASA/Cl group when compared with not treated 4T1 control mice (471.4 vs 219.6 pg·ml·10^6^PLT for control and ASA-Cl mice, respectively, n=7-12, P=0.0010 in basal conditions as well as after 60 minutes of mechanical stimulation (stirring): 10024 vs 1017.0 pg·ml·10^6^ PLT for control and ASA-Cl-treated mice, respectively, n=7-12, P=0.0015) (Figure 2E). Effectiveness of platelet inhibition by ASA-Cl treatment was also confirmed by decreased tendency of platelets to form aggregates in the circulation when compared to control 4T1 mice: no difference between control 4T1 and 4T1+ASA/Cl groups was observed with respect to the number of smaller objects <2.5 μm, but the number and size of larger platelet aggregates > 10 μm were significantly lower in ASA-Cl-treated mice (Figure 2F).

### Effects of aspirin and clopidogrel on development of vascular mimicry in the primary tumour in orthotopic model of metastatic 4T1 breast cancer

**Figure 3:**
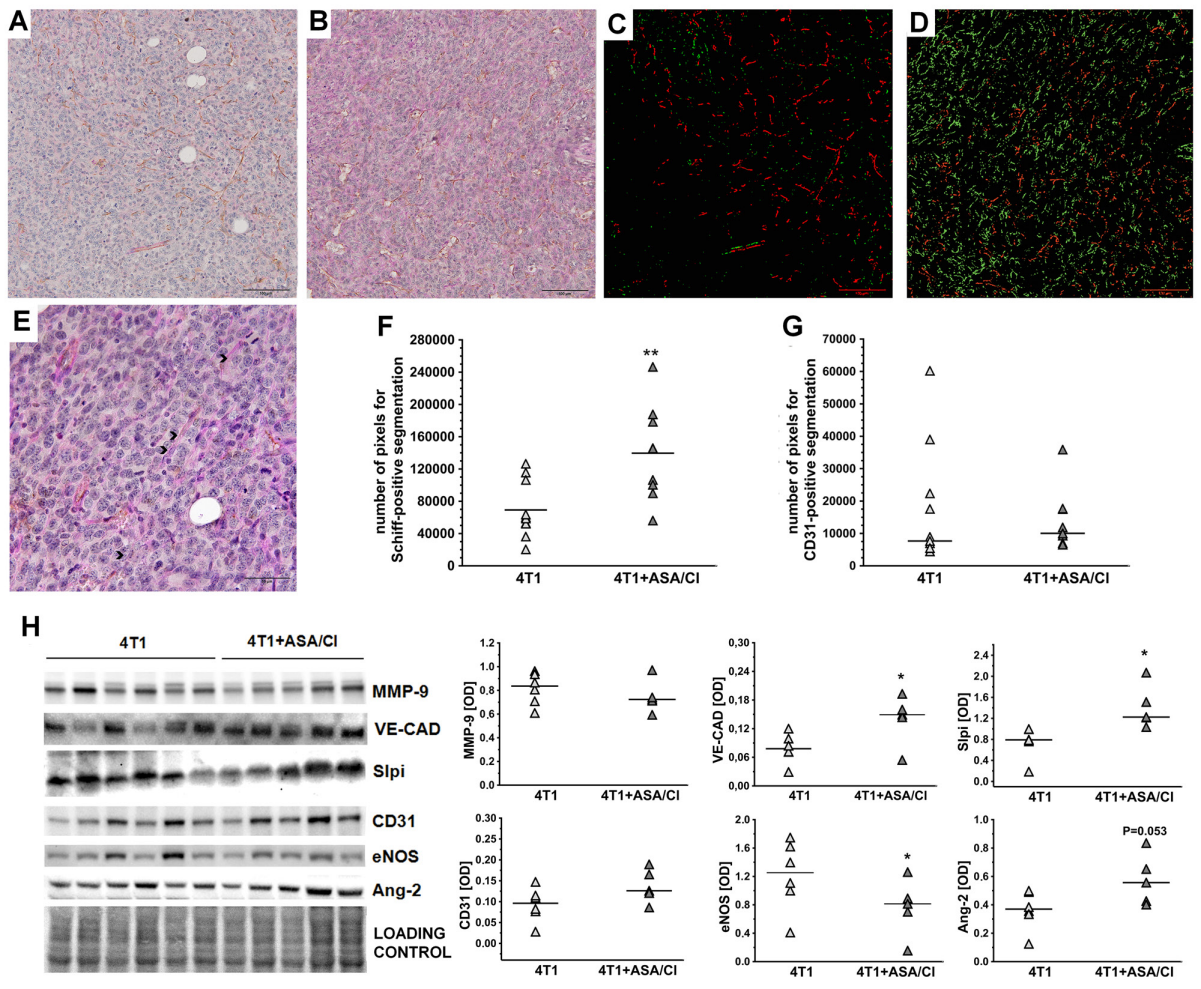
Effects of dual anti-platelet therapy with aspirin and clopidogrel on VM in the primary tumour in orthotopic metastatic breast cancer model Data are presented as mean of data without outliers **(F)**; n =10 for control 4T1 mice, n=9 for ASA+Cl-treated 4T1 mice and **(G)** as median of data without outliers; n=11 for control 4T1 mice and n=9 for ASA+Cl-treated 4T1 mice. Primary tumours of 4T1 and 4T1+ASA/Cl mice were cut into slices and stained to visualize basement membranes (Periodic Acid Schiff staining, PAS) and endothelium-lined blood vessels (CD31). Subsequently, ten randomly chosen eyefields near the regions of hypoxia were photographed for each mouse and subjected to segmentation using *Ilastik* software to assess the number of PAS+/CD31- pseudovessels and PAS+/CD31+ endothelium-lined vessels, as described in *Materials and Methods*. The representative photographs of VM in primary tumours of 4T1 and 4T1+ASA/Cl groups are given in **(A and B)** and the results of their segmentation are given in **(C and D)**, respectively. For (C and D), green indicates PAS+/CD31- pseudovessels and red indicates PAS+/CD31+ endothelium-lined vessels. In, **(E)** higher magnification of the data presented in (B) is shown with black arrows pointing to the PAS+/CD31- pseudovascular channels perfused by RBC. The mean number of pixels indicating pseudovessels (F) and endothelium-lined vessels (G) was counted with the use of Image J. The data were analyzed with either two-sided Student *t* test (F) test or Mann-Whitney *U* test (G) depending on the normality of distribution and variance homogeneity (tested with *F* test); the symbol ^**^ indicates statistical significance at P<0.01. For determination of VM molecular markers, primary tumours were excised, homogenized and individual samples (n=6 form 4T1 and n=5 from 4T1+ASA/Cl group) were run on polyacrylamide gels **(H)**. Subsequently, they were processed as described in *Materials and Methods*. Densytometric data [OD] normalised to the total protein are presented as median of the data without outliers. Homogeneity of variances and normality of densytometric data distribution were confirmed by F and KS normality tests, respectively, and the data were analysed with two-sided two-sided Student *t* test. The symbol ^*^ indicates statistical significance at P<0.05.

**Figure 4:**
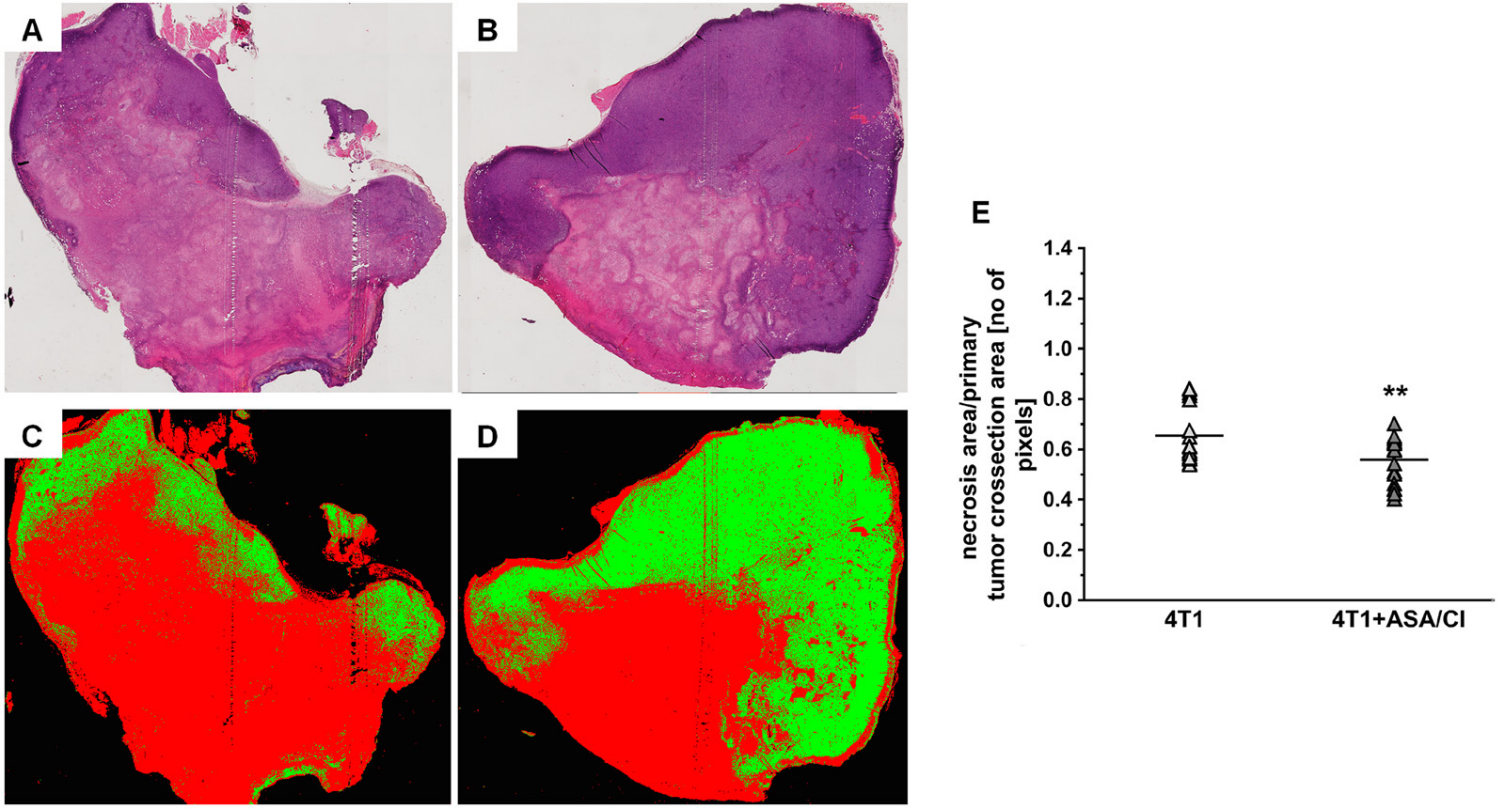
Effects of dual anti-platelet therapy with aspirin and clopidogrel on necrosis area in the primary tumour in orthotopic metastatic breast cancer model Data in **(E)** are presented as mean of data without outliers; n=20 for control 4T1 mice and n=18 for ASA+Cl-treated mice. Primary tumours of 4T1 and 4T1+ASA/Cl mice were cut into slices and stained with H&E to assess the relative necrotic area, as described in *Materials and Methods*. Subsequently, the total tumour cross-section area was scanned with a BX51 microscope equipped with virtual microscopy system dotSlide (Olympus, Japan) and subjected to segmentation in *Ilastik* software to assess the relative necrotic area. The representative photographs for primary tumours of 4T1 and 4T1+ASA/Cl groups are given in **(A and B)**, and the results of their segmentation are given in **(C and D)**, respectively. For (C and D), red indicates necrotic area while green indicates viable tumour tissue. The mean number of pixels representing necrotic and viable tumour tissue ± SD was counted in Image J (E). Based on the normality of distribution and variance homogeneity (*F* test), the data in (E) were analysed with two-sided Student *t* test. The symbol ^**^ indicates statistical significance at P<0.01.

In 4T1+ASA/Cl group, the number of PAS-positive and CD31-negative pseudovessels in primary tumours was higher compared to control 4T1 mice (69310±35033 pixels for 4T1 mice vs 139608±58524 pixels for 4T1+ASA/Cl mice, n=9-10, P=0.005) (Figure 3A-3F), compatible with vascular mimicry (VM) in the primary tumours induced by ASA+Cl treatment, with no difference in the number of PAS-positive and CD31-positive endothelium-lined vessels (Figure 3A-3D and 3G). These results indicated a higher number of pseudovessels in ASA+Cl-treated mice without any difference in the number of blood vessels between ASA+Cl-treated and control 4T1 mice. Induction of VM in primary tumours of 4T1+ASA/Cl mice was confirmed by higher expression of VE-cadherin (VE-CAD) and secretory leukocyte protease inhibitor (Slpi) (Figure 3H) with no change in MMP-9, all reported to be markers of VM [24, 22, 25, 26]. Blood vessels in primary tumours of 4T1+ASA/Cl group were dysfunctional, as evidenced by low expression of the endothelial isoform of nitric oxide synthase (eNOS) and higher expression of Angiopoetin-2 (Ang-2) (Figure 3H). Induction of VM by ASA+Cl treatment was associated with lower relative necrotic area in the primary tumours of mice receiving the ASA+Cl anti-platelet therapy (0.65±0.1 for 4T1 mice vs 0.56±0.09 for 4T1+ASA/Cl mice, n=18-20, P=0.006) (Figure 4A-4E).

### Effects of aspirin and clopidogrel on pro-angiogenic phenotype of platelets in the orthotopic metastatic 4T1 breast cancer in mice

**Figure 5:**
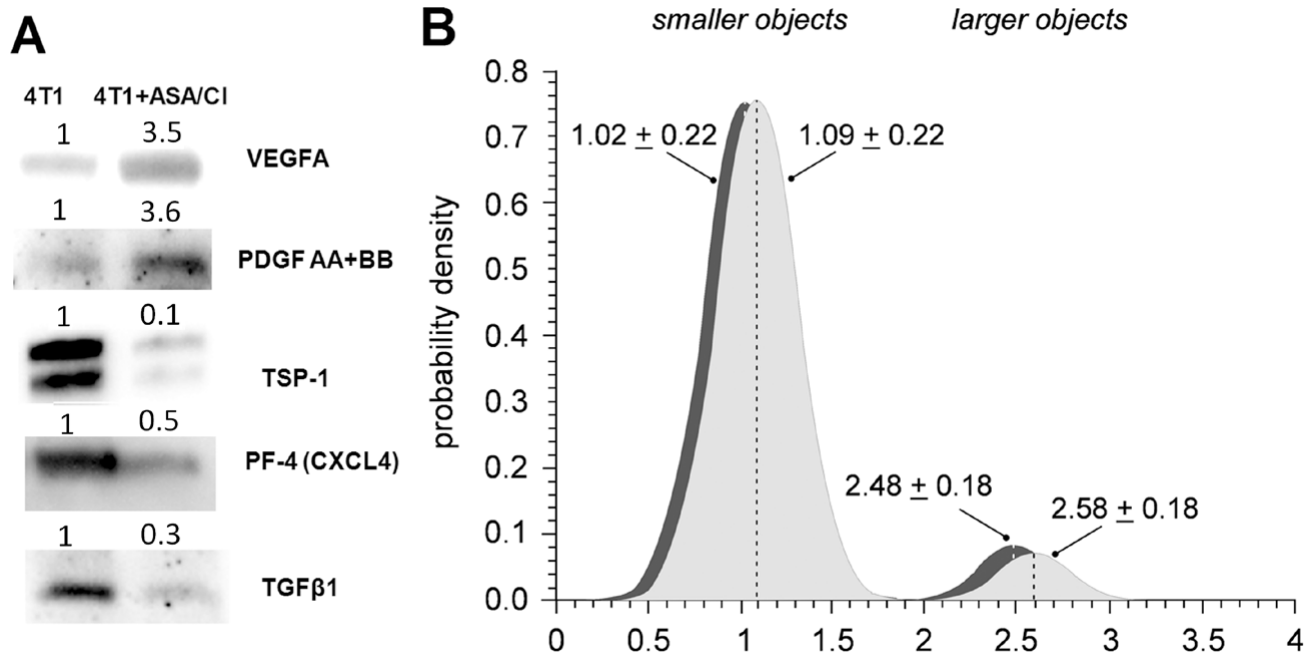
Effects of dual anti-platelet therapy with aspirin and clopidogrel on platelet size and pro-angiogenic phenotype in orthotopic metastatic breast cancer model **(A)** To investigate the angiogenic phenotype of platelets, isolated platelets were lysed and pooled together (n≥6) to obtain sufficient protein amount for WB analysis. Equal amounts of pooled samples were separated on the SDS-PAGE, transferred and probed for antiangiogenic as well as proangiogenic proteins stored in platelet α-granules: antiangiogenic thrombospondin-1 (TSP-1), platelet factor 4 (PF4/CXCL4), transforming growth factor β1 (TGFβ1) and proangiogenic vascular endothelial growth factor (VEGF(A)) and platelet-derived growth factor (PDGF AB +BB). The images in (A) present the fold change of each protein of interest in 4T1+ASA/Cl group vs control 4T1 group. Equal protein loading was controlled after electrophoresis and transfer for all gels and membranes using a stain free-technique, as described in *Materials and Methods*. **(B)** Data are presented as mean ± SD or median and IQR; n= 7 for control 4T1 mice (light grey) and n = 7 for ASA+Cl-treated 4T1 mice (dark grey). The subpopulation of normoplatelets presented in Figure 2F was decomposed into two clusters, representing smaller (subpopulation 1) and larger (subpopulation 2) platelets. Object sizes were estimated from the standard curve assigned with FSC calibration beads (size range of 1 μm to 15 μm). The contributions of smaller and larger objects in the population of normoplatelets were 92.9% and 7.1% in 4T1 controls vs. 92.2% vs. 7.8% in 4T1 treated with ASA+CL (P< 0.003 for larger objects: 7.1; 6.5-7.5% in control vs. 7.8; 6.5-9.3% in 4T1+ASA/Cl mice, bootstrap estimate of the one-sided Mann-Whitney *U* test, 1000 iterations).

Pro-angiogenic phenotype of platelets decreases with platelet age, since senescence of platelets is associated with their loss of function and smaller size [27, 28]. Therefore, “young” platelets contain more proangiogenic proteins and are bigger in size, whereas for “old” platelets the opposite is observed. As shown in Figure 5A, platelet phenotype in 4T1+ASA/Cl mice was more pro-angiogenic, since they contained more proangiogenic VEGFA and PDGF AB +BB and less antiangiogenic TSP-1, PF4/CXCL4 and TGFβ1 when compared with platelets isolated from control 4T1 mice. Moreover, despite the similar number of smaller “old” platelets in the circulation of 4T1+ASA/Cl mice and control 4T1 mice, the number of large “young” platelets in 4T1+ASA/Cl mice was higher compared with control 4T1 mice (Figure 5B), indicating thrombopoesis in response to platelet inhibition with ASA+Cl.

### Effects of aspirin and clopidogrel or platelet-depleting antibody on pulmonary metastasis after intravenous injection of 4T1 breast cancer cells

**Figure 6:**
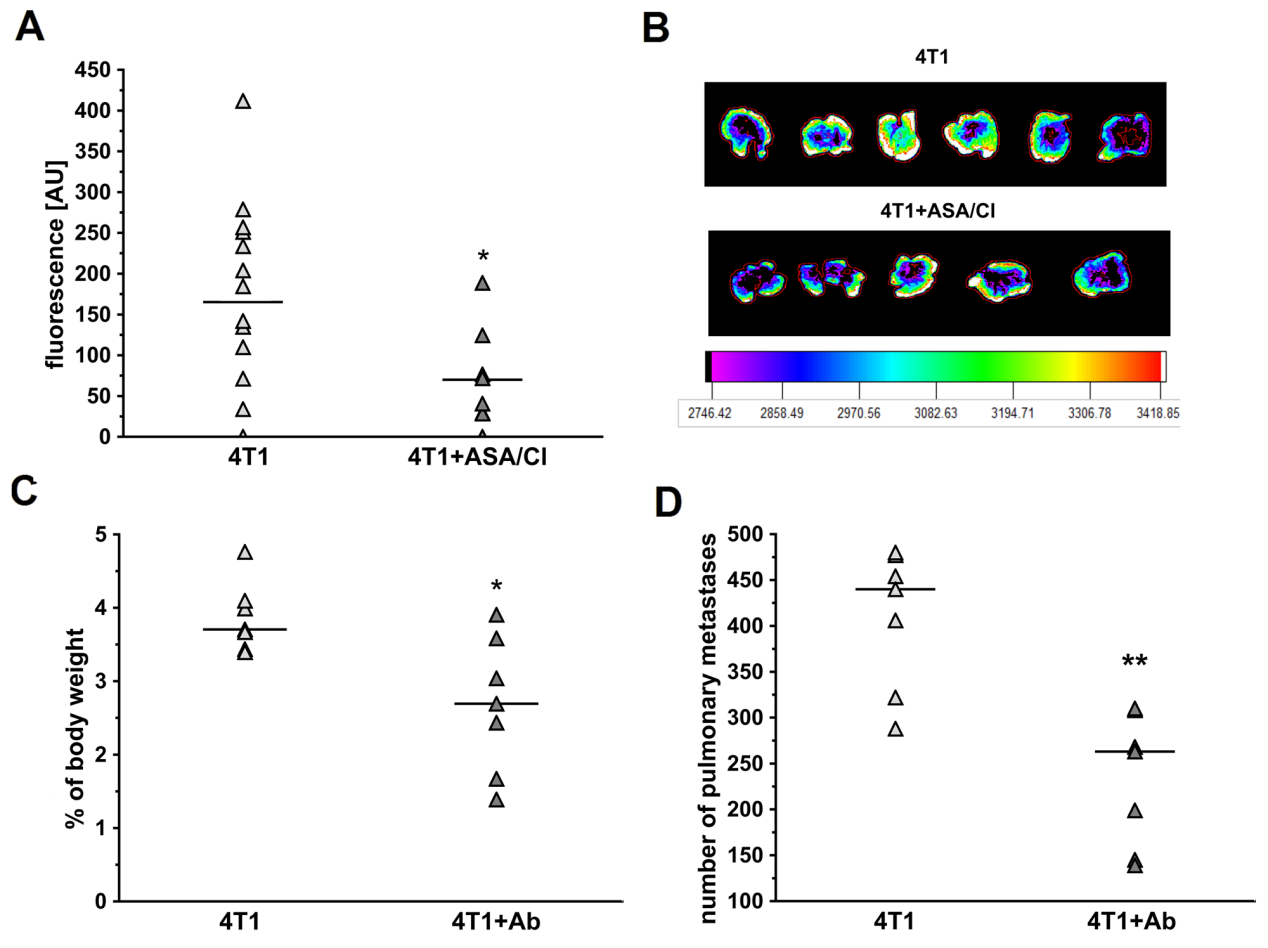
Effects of dual platelet inhibition with aspirin and clopidogrel and platelet depletion on pulmonary metastasis in intravenous murine breast cancer model For (A), the results are presented as mean of data without outliers; n=14 for control 4T1 mice, n=8 for ASA+Cl-treated 4T1 mice, in the case of (C, D) the results are presented as median of data without outliers; n=7 for control 4T1 and ASA+Cl-treated mice. **(A, B)** Mice were injected intravenously (i.v.) with 2×10^5^ of 4T1-luc2td-Tomato cells and were randomly assigned either to control not-treated group (4T1) or the group that received dual anti-platelet therapy (ASA+Cl) (aspirin and clopidogrel 1/1 mg · kg^-1^ of body weight, respectively) (4T1+ASA/Cl) 45 min prior to cancer cell inoculation. Five hours before cancer cell transplantation, mice were injected intraperitoneally (i.p.) with 1 mg · kg^-1^ of LPS. Mice were sacrificed 24 hours after cancer cell transplantation, lungs were collected and their fluorescence was analyzed, as described in *Materials and Methods*. **(C, D)** Mice were injected with platelet-depleting antibody 15 min before 4T1 breast cancer cell injection (4T1+Ab) or did not receive the antibody (4T1). After 13 days they were euthanized, their lungs were excised and weighed and lung weight was expressed as the % of body weight. The number of pulmonary metastases was counted on the lobe surface under the magnifying glass after dissection of formaldehyde-fixed lungs. Based on the normality of distribution and variance homogeneity (*F* test), the data were analyzed with Mann-Whitney *U* test. The symbols ^*^ and ^**^ indicate statistical significance at P<0.05 and P<0.01, respectively.

To investigate whether anti-platelet therapy with ASA+Cl would decrease pulmonary metastasis in the absence of primary tumour, mice were injected i.v. with ASA+Cl and then with 4T1-ln2-tdTomato cells. As shown in Figure 6A and 6B, combination of ASA and Cl decreased pulmonary metastases after i.v. injection of 4T1-ln2-tdTomato cells (assessed as lung fluorescence intensity: 165±118 a.u. in control 4T1 mice vs 70±61 in 4T1+ASA/Cl mice, n=8-14, P=0.0342 by bootstrap-boosted unpaired Student’s *t* test). Similarly, depletion of platelets with antibody inhibited metastasis after i.v. injection of 4T1 cells (the difference in lung weight: 3.7; 3.4-4.1% of body mass of control 4T1 mice vs 2.7; 1.7-3.6% of body mass of 4T1+Ab mice, n=7, P=0.017, and the number of lung metastases in control 4T1 mice 440.0;322.0-477.0 vs 263.0; 145.0-308.0 for 4T1+Ab mice, n=7, P=0.002, Figure 6C and 6D). Lower number of pulmonary metastases after platelet depletion was also associated with improved systemic endothelial function in 4T1+Ab mice, as evidenced by higher production of NO in aorta of 4T1+Ab group as compared with 4T1 mice (Supplementary Figure 2).

## DISCUSSION

In the present work, we demonstrated that dual antiplatelet therapy with aspirin and clopidogrel (ASA+Cl) given to 4T1 breast cancer-bearing mice after orthotopic inoculation of cancer cells effectively inhibited ADP-and TXB_2_-dependent responses in platelets, inhibited pro-thrombotic phenotype of platelets (Figure 2) but stimulated vascular mimicry (VM) in the primary tumour (Figure 3), resulting in accelerated progression of the disease and decreased mice survival (Figure 1). In contrast, in mice with 4T1 cells injected intravenously and, thus, without a primary tumour, inhibition of platelets with ASA+Cl or platelet depletion with anti-platelet antibody significantly reduced pulmonary metastasis (Figure 6). Our results demonstrate for the first time, that platelet-dependent mechanisms inhibited by dual antiplatelet therapy with aspirin and clopidogrel promoted cancer malignancy and VM in the presence of primary tumour but afforded protection against pulmonary metastasis in the absence of primary tumour.

For many years, platelet inhibition was considered to slow down the progression of cancer [4, 5, 29–31]. However, in the last decade evidence has emerged suggesting that chronic platelet inhibition could paradoxically promote progression of malignant diseases in initially cancer-free patients [8, 9, 11, 21]. This observation was confirmed by the DAPT clinical trial: long-term platelet inhibition by thienopyridines treatment, antagonizing P2Y_12_ receptors such as prasugrel or clopidogrel, was associated with statistically significant increase in the incidence of new solid cancer cases and the ratio of cancer deaths among initially cancer-free cardiovascular patients [14]. In view of data presented in this study, we postulate that these adverse effects of thienopyridines might be related to induction of VM and formation of pseudovascular circulation in latent tumours, promoting their malignancy.

Vascular mimicry, discovered by [32], is a very common phenomenon in various malignant cancers, including breast cancer [22, 33, 34], and is always associated with increased malignancy of the disease, poor patient prognosis and decreased survival [32]. Indeed, we demonstrated here that increased mortality of mice orthotopically injected with 4T1 breast cancer cells receiving dual anti-platelet therapy based on aspirin and clopidogrel throughout the progression of the disease was associated not only with more pronounced VM in their primary tumours (Figures 3A-3F), but also with the increase of other negative markers of cancer malignancy: TGFβ1, COX-2 and PGI_2_S levels in the lungs (Supplemetary Figure 1) [35–37] as well as with an increased systemic NO bioavailability (Table 1) [38], which could indicate more pronounced cancer-associated immunosuppression in this cancer model since myeloid-derived suppressor cells (MDSCs) are known to produce large quantities of NO in an arginase- dependent way [39]. Furthermore, despite effective attenuation of platelet pro-thrombotic function (Figure 2), dual anti-platelet treatment changed the balance of pro/anti-angiogenic phenotype of platelets towards more pro -angiogenic (Figure 5A) that could be at least partially explained by the stimulation of thrombopoesis by aspirin-clopidogrel treatment. Pro -angiogenic phenotype of platelets could contribute to cancer progression [2] as well as to VM in aspirin-clopidogrel treated mice orthotopically injected with 4T1 breast cancer cells.

Apart from the well-known role in coagulation, platelets contain a plethora of growth factors that safeguard blood vessel integrity and promote survival of endothelial cells [40]. We suggest that deficiency of platelet-derived growth factors in mice subjected to dual anti-platelet therapy with ASA+Cl could contribute to dysfunction of the primary tumour microvasculature what could drive VM by VEGF/VEGFR1-dependent pathway [41, 42] to support tumour perfusion. Indeed, the intratumour area of necrosis was smaller in ASA+Cl-treated mice (Figure 4), suggesting better tumour perfusion concomitantly with induction of VM, as evidenced by higher number of PAS+CD31- pixels (Figure 3A-3F) and higher levels of VE-CAD and Slpi considered as molecular markers of VM [22, 25, 26] (Figure 3H). Interestingly, the number of endothelium-lined blood vessels was similar in the primary tumours of ASA+Cl-treated and control 4T1 mice (Figure 3A-3E, 3G-3H) though they were more dysfunctional in mice with inhibited platelets, as evidenced by lower expression of eNOS (a marker of “healthy” endothelium) in the primary tumour. Also Ang-2, known as the vascular destabilizing factor, tended to be higher in ASA+Cl-treated mice compared to its expression in the primary tumours of control 4T1 mice (Figure 3H). Altogether, our results demonstrate that in ASA+Cl-treated mice VM was activated, concomitantly with more pronounced microvascular dysfunction in primary tumours. These results stay in line with the emerging role of platelets in the regulation of microvascular integrity that could be of particular importance in the microvascular network of primary tumour [43, 44]. In fact, induction of thrombocytopenia consistently resulted in massive bleeding in and surrounding the tumour, without affecting vascular integrity elsewhere [41, 44].

In the present work, the number of secondary nodules or the relative area of metastatic foci in lungs was not higher in ASA+Cl-treated mice (Table 1). The expression of metalloproteinases (MMPs), von Willebrand factor (vWF) and pro-angiogenic VEGF and EphA1 was also not higher in the lungs of ASA+Cl-treated mice, while endothelium-protective Slit2/ROBO4-dependent signaling known to prevent endothelium permeability in the lungs was activated (Supplementary Figure 1). This discrepancy between induction of VM in the primary tumour and the lack of increased pulmonary metastasis might seem intriguing. It was previously shown that the platelet depletion increased metastatic spread to the lungs in this model of breast cancer [22, 23]. However, if platelet inhibition would lead to more efficient intravasation of 4T1 cells in ASA+Cl-treated mice due to more pronounced VM in the primary tumour, it could be efficiently counterbalanced by aspirin plus clopidogrel treatment lowering metastatic capacity of circulating 4T1 cells (i.e. aspirin inhibited thromboxane A_2_ generation by platelets and 4T1 cells what lowered metastatic capacity of the latter [45]). Indeed, if 4T1 cells were injected intravenously, both platelet inhibition and platelet depletion reduced pulmonary metastasis (Figure 6) similarly, as shown previously [22], clearly suggesting that inhibition of platelets by aspirin and clopidogrel inhibited extravasation and pulmonary metastasis of 4T1 breast cancer cells. These data confirm that platelet inhibition indeed decreases extravasation but, if the primary tumour is present, these beneficial effects can be overridden by induction of VM what could lead to more efficient intravasation of cancer cells.

Therefore, better understanding of distinct platetet-dependent mechanisms regulating extravasation and intravasation of cancer cells is fundamental for effective and safe anti-platelet therapy.

To summarize, we demonstrated for the first time that, dual antiplatelet therapy with aspirin and clopidogrel promoted cancer malignancy and VM in the presence of primary tumour. On the other dual antiplatelet therapy with aspirin and clopidogrel afforded protection against pulmonary metastasis in the absence of primary tumour. Further studies are needed to define the details of platelet-dependent mechanisms promoting cancer malignancy and VM in the presence of primary tumour and those affording protection against pulmonary metastasis in the absence of primary tumour. Our results suggest that long-term platelet inhibition with dual antiplatelet therapy based on aspirin and clopidogrel, though affording unequivocal cardiovascular benefits, could pose a significant hazard when given to a patient with undiagnosed and untreated malignant cancer prone to undergo VM.

## MATERIALS AND METHODS

### Animals

Female Balb/C mice aged 7-11 weeks were used for the experiments (Charles River Laboratory, Germany or Medical Centre Bialystok, Poland). Throughout the experiment, animals were housed 5-6 per cage, in a temperature-controlled environment (22–25°C), maintained on a 12-hour light/day cycle and given unlimited access to food (Zoolab, Krakow, Poland) and water. Animals were euthanized by i.p. injection of ketamine and xylazine, 100 and 10 mg · kg^-1^, respectively. All experimental procedures involving animals were accepted by the First Local Ethical Committee on Animal Testing at the Jagiellonian University (Krakow, Poland), permit no: 140/2013, the Local Ethical Committee on Animal Testing at the Institute of Immunology and Experimental Therapy (Wrocław, Poland) no: 78/2015, and the Second Local Ethical Committee on Animal Testing in the Institute of Pharmacology, Polish Academy of Sciences (Krakow, Poland), permit no: 41/2017. ***Orthotopic injection***: 120 mice were randomly divided into control group (80 mice; 4T1) or treated group that received combined anti-platelet therapy: aspirin and clopidogrel (acetylsalicylic acid was obtained from Sigma Aldrich (A5376) whereas clopidogrel was extracted from Plavix (Sanofi) by Lodz University of Technology), 12 mg· kg^-1^ of body weight^-1^ · 24h^-1^ each (40 mice, 4T1+ASA-Cl). Drugs were pre-mixed with the standard chow diet and the food intake was monitored throughout the experiment. All mice were injected orthotopically with 1 × 10^4^ 4T1 murine breast cancer cells into one right thoracic mammary gland and received anti-platelet therapy starting from the 11^th^ day after 4T1 cell injection, when the primary tumours were palpable in all mice. The anti-platelet therapy was continued until the end of the experiment. Animals were euthanized in the 5^th^ terminal week of the disease. Animals from each group that died before their planned euthanasia were used to calculate survival in both groups. ***Intravenous injection***: For the 24-hour model, 22 Balb/C mice were injected intravenously (i.v.) with 2×10^5^ of 4T1-luc2td-Tomato cells following intraperitoneal injection (i.p.) of LPS at the dose of 1 mg · kg^-1^ of body weight 5 h before cancer cell inoculation. Aspirin and clopidogrel (both at a dose of 1 mg · kg^-1^ of body weight) were administered intravenously 45 min before cancer cell inoculation (8 4T1+ASA/Cl mice) or saline (14 control 4T1 mice). The fluorescence of 4T1-luc2td-Tomato cells in the lungs was measured 24 h after cancer cell injection using an *in vivo* MS FX PRO system (Carestream Health INC., USA). Images were analyzed with Carestream MI SE software (Carestream Health INC., USA). The intensity of the fluorescence signal is presented as the sum intensity of the region of interest and expressed in arbitrary units. For the 2-week model, 20 Balb/C mice were injected i.v. with 7.5×10^4^ 4T1-luc2td-Tomato cells following i.v. injection of platelet-depleting antibody at the dose of 4 μg · g^-1^ of body weight (Emfret Analytics) (Ab mice) 15 min before cancer cell inoculation. It was confirmed that the number of circulating platelets was reduced by 75% at that time (data not shown). Lung weight was measured and the number of pulmonary metastases was counted after mice euthanasia on the 13^th^ day after 4T1 cell injection.

### Cell culture

The mouse mammary adenocarcinoma 4T1 cells were obtained from the American Type Culture Collection (ATCC, USA) and 4T1-luc2-tdTomato cell line stably expressing the firefly luciferase gene and tdTomato fluorescent protein was obtained from Caliper Life Sciences Inc. (USA). 4T1 cells were cultured in RPMI 1640-Glutamax medium (Sigma-Aldrich, Poland) supplemented with 10% fetal bovine serum (Gibco, Thermo Fisher Scientific, Poland), 1.0 mM sodium pyruvate (Sigma-Aldrich, Poland) and antibiotic antimycotic solution (100 units/mL penicillin and 100 μg/mL streptomycin, 25 μg/mL amphotericin B) (Sigma-Aldrich, Poland). Cells were cultured at 37°C in a humidified atmosphere containing 5% CO_2_. For inoculations, only 4T1 cells at the 2^nd^ passage were used. Prior to the transplantations, 4T1 cells were detached using Accutase solution (Sigma-Aldrich, Poland), centrifuged (300 g, 4°C, 5 min), counted, suspended in Hank’s Balanced Salt Solution (HBSS, IIET, Poland) at the appropriate count and inoculated into the mammary gland of female Balb/C mice or injected intravenously as described above. All cell cultures were routinely tested for *Mycoplasma* contamination.

### Monitoring of primary tumour growth and pulmonary metastasis development

In the orthotopic model, the progression of breast cancer was monitored throughout the 5 weeks after inoculation of 4T1 breast cancer cells into the mammary fat pads of Balb/C mice by measuring their body weight and primary tumour volume with calipers once a week as described previously [46]. After euthanasia, primary tumours, lungs and spleens were excised, weighed and fixed in formalin for histological and immunohistochemical staining or frozen in liquid nitrogen for Western blot analysis. The number of pulmonary metastases was counted on lung lobes under the magnifying glass and on lung cross-sections stained with hematoxilin and eosin (H&E). Concomitantly, the relative metastatic area was measured and presented as the percentage of the cross-section area of the lung lobes. For 2-week intravenous model, body mass of mice was measured a week before 4T1 cell injection, at the time of injection and then once a week until mice were euthanized. After euthanasia, lungs were excised, weighed and fixed in formalin and the number of pulmonary metastases was counted on the lung lobes under the magnifying glass.

### Flow cytometry

Blood samples were collected on citrate from the right heart ventricle and processed as reported earlier [47]. The samples designated for flow cytometry measurements were diluted with saline, washed with Tyrode buffer and incubated with anti-mouse PE-conjugated antibody directed against the active form of GPIIb/IIIa or P-selectin for determination of their platelet surface expression and anti-mouse FITC-labeled fibrinogen or von Willebrand (vWF) factor antibodies for determination of their binding on the platelet surface. Basal activation of circulating platelets and their ADP-dependent reactivity were evaluated on the basis of the measured expressions/binding of surface membrane antigens in samples supplemented with PBS or ADP to a final concentration of 20 μM, respectively. Platelets were identified by their forward- and side-scatter characteristics and were gated on the basis of the expression of platelet-specific antigen CD41/61. Isotype matched FITC- or PE-conjugated control antibodies were used to assess non-specific binding. Flow cytometric analyses of platelet activation was performed using flow cytometry software (LSRII and FACS/Diva ver. 6.0, respectively, Becton Dickinson, Oxford, UK). Measurements were made under a logarithmic gain and at least 10,000 events were collected. Appropriate colour compensation was determined in samples singly stained with either FITC-conjugated anti-CD41/61 or PE-conjugated anti-CD41/61. Unstained platelets were used to establish the level of autofluorescence that was set to fall within the first log order of brightness for each fluorescence channel. Suitable isotype controls were used as appropriate to set up the background noise at less than 1%. Events appearing above the background level were then recorded. Results were presented as the percentage of activation marker-positive events in the platelet population.

### *Ex vivo* assay for TXB_2_ generation in stirred blood

Blood samples were diluted with saline (5 times) and stirred in alternating directions in 1ml cuvettes with disposable, siliconized stir bars (Chrono-Log, US) for one hour in 37°C (1500rpm; spinning time in one direction: 3s; acceleration/deceleration: 20000 rpm·s^-1^) in a specially-designed Xyzyk apparatus (Xyzyk Co, Poland), as described previously [48]. To determine basal and mechanically-stimulated *ex vivo* TXB_2_ generation in diluted blood, samples were taken on aspirin (500 μM) at the 2^nd^ and 60^th^ minute of *ex vivo* stirring of blood samples and centrifuged to obtain plasma (3000xg, 12 min, 4°C). TXB_2_ concentration in plasma was measured using an enzyme-linked immunosorbent assay ELISA kit (Enzo Life Sciences).

### Western blotting

Platelets were isolated as described by [49]. Blood samples from mice were collected on 3.8 % sodium citrate. The samples were centrifuged at 100 G for 10 min to obtain PRP. Subsequently, platelets were pelleted at 600 G for 10 min. Platelet poor plasma (PPP) was discarded and the pellet containing platelets was suspended in Tyrode buffer and pelleted again at 600 G for 10 min. Subsequently, platelet pellet was put on ice and suspended in the lysis buffer containing protease and phosphatase inhibitors. Protein concentration was measured before sample pooling (BCA assay). Because of the limited protein content of platelet samples, the Western blot experiments were carried out on the pooled samples. The resting state of isolated platelets was confirmed by measuring their ADP-induced reactivity by flow cytometry. Lungs and primary tumours were homogenized for protein extraction with protease and phosphatase inhibitors. Protein concentration was measured with bicinchoninic acid assay (BCA assay). Subsequently, the samples from six mice in each experimental group were pooled together in such a way that an aliquots corresponding to equal amounts of protein from each sample were suspended in equal volumes for all mice in the group, in order to ensure equal representation of each individual sample in the pooled specimen. After addition of loading buffer, samples were heated at 96°C for 5 min and then stored at -80°C. Equal amount of protein from each pooled sample was loaded and run on the gel, transferred to nitrocellulose or PVDF membrane, blocked with 5% dry milk and incubated with the appropriate primary antibodies directed against the following antigens: MMP-2 (ab19167), MMP-9 (ab19016), Ang-1 (ab8451), TSP-1 (ab85762), TGFβ1 (ab155264), VEGF(A) (ab68334), PDGF AB+BB (ab34074), vWF (ab9378), Slit2 (ab134166) were from Abcam, UK; MMP-14 was from Sigma Aldrich (sab4501901); Slpi (sc-374575), EphA1 (sc-377362), PGI_2_S (sc-20933) and VE-CAD (sc-6458) were from Santa Cruz Biotechnology (TX, US); CD31 was from Novus Biologicals (NBP1-71663H); COX-2 was from Cayman Chemicals (aa584-598); eNOS was from BD Transduction Laboratories (610296); Ang-2 was from Thermo Fisher Scientific (PA5-27297); PF4 was from R&D Systems (AF595-SP); ROBO4 was from Biorbyt (orb101060). The appropriate HRP-conjugated secondary antibodies were from Santa Cruz Biotechnology (sc-2020, sc-2004 and sc-2005). For primary antibodies/antigens producing ambiguous chemiluminescent signal, the experiments were repeated at least twice. Equal protein loading was controlled after electrophoresis and transfer for gels and membranes, respectively, using a stain free-technique provided by Bio-Rad [50]. In case of primary tumours, the levels of selected antigens were probed not only on pooled samples but also on individual samples to confirm differences obtained in pooled samples.

### Histology

Spleens, lungs and primary tumours were fixed in formalin, paraffin-embeded and cut into 5 μm slices. Spleen cross-sections were stained with H&E and the number of megakaryocytes was counted for each mouse in ten random eyefield microphotographs of the spleen that were performed in such a way that the edge of the spleen was visible on each microphotograph, since most of the megakaryocytes resided in the spleen periphery. The mean number of megakaryocytes in one eyefield was counted for each mouse. Lung cross-sections were stained with H&E to count the number of metastatic foci and measure the relative metastasis area vs lung cross-section area, as described above. Primary tumour cross-sections were stained with H&E to assess the relative necrosis area, as reported by [51], and with combination of Periodic Acid Schiff staining (PAS) and anti-CD31 (ab28364, Abcam, UK) to assess the number of PAS+/CD31- pseudovessels and PAS+/CD31+ endothelium-lined blood vessels, as reported previously [22]. All images were scanned with a BX51 microscope equipped with virtual microscopy system dotSlide (Olympus, Japan). Image segmentation was performed using *Ilastik* (developed by the Ilastik team, with partial financial support by the Heidelberg Collaboratory for Image Processing, HHMI Janelia Farm Research Campus and CellNetworks Excellence Cluster).

### Measurement of blood cell count and nitrite, nitrate in plasma and HbNO in blood

The blood cell count was measured with an animal blood counter Vet abc (Horiba Medical, France). The samples designated for plasma extraction were centrifuged at 1000 x g/10 min/4 °C and plasma was aliquoted for measurement of NO_2_^-^ and NO_3_^-^ concentrations with ENO-20 NOx Analyzer (Eicom Corp., Kyoto, Japan). RBC were used for determination of nitrosylhemoglobin (HbNO) levels. The detection of HbNO was based on EPR spectra of the samples (erythrocytes obtained from whole blood by a 5 min centrifugation at 1000 x g in 4 degrees Celsius) recorded in liquid nitrogen (77 K) using a Bruker EMX Plus spectrometer operating at X-band using a 1041HS resonator. The following conditions were used: center field at G=2, microwave power 15.89 mW; time constant of 81.92 ms; modulation frequency 100 kHz; modulation amplitude 5 G; scan time 20.48 s; scan width 200 G. For each sample, 30 individual scans were averaged. The level of microwave power has been determined from the power saturation curve to avoid saturation of the HbNO signal. For EPR signals values of amplitude of the second hyperfine line of nitrosylhemoglobin EPR spectra was used to calculate content of HbNO and the signal was normalized to sample weight and expressed in arbitrary units. The overlaying free radical signals at G = 2 were deconvoluted to enhance the HbNO signal assessment using an in-house algebraic process.

### Statistical analysis

Data were presented as mean or median of the data without outliers (determined by Grubbs’ test) depending on data distribution (tested with D’Agostino test and Pearson omnibus normality test) and homogeneity of variances (tested with Brown-Forsythe’s test, *F* test or Barlett’s test). Some variable non-conforming with normal distribution and/or variance homogeneity were Box-Cox transformed and analysed with parametric tests, otherwise they were analysed with nonparametric inference tests. Only post-hoc P values < 0.05 were considered significant. The survival analysis was performed with the use of the Kaplan-Meier curves and the generalised Wilcoxon (Peto-Prentice) test.

## SUPPLEMENTARY MATERIALS FIGURES



## Abbreviations

ASA: aspirin
Cl: clopidogrel
VM: vascular mimicry
HbNO: nitrosylhemoglobin
H&E: haematoxilin and eosin staining
PAS: Periodic Acid Schiff staining
