# Supplementary material for: Dual antiplatelet therapy with clopidogrel and aspirin increases mortality in 4T1 metastatic breast cancer-bearing mice by inducing vascular mimicry in primary tumour
Source: Oncotarget. 2018 Apr 3;9(25):17810–24. doi: 10.18632/oncotarget.24891 (PMC5915156; doi:10.18632/oncotarget.24891)
Supplement: Supplementary file 1 [file oncotarget-09-17810-s001.pdf]

Dual antiplatelet therapy with clopidogrel and aspirin increases mortality in 4T1 metastatic breast cancer-bearing mice by inducing vascular mimicry in primary tumour

SUPPLEMENTARY MATERIALS

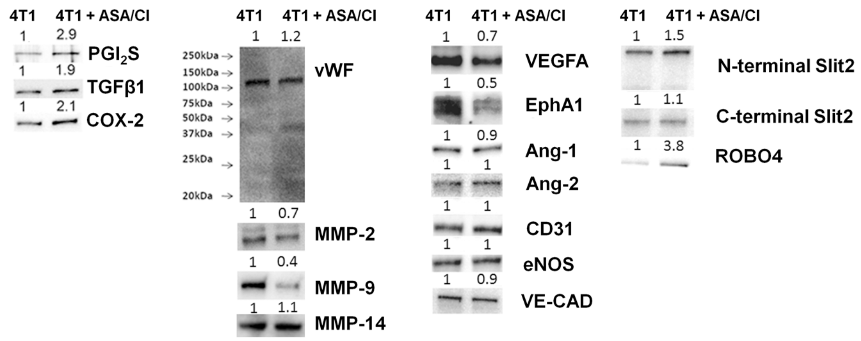

Supplementary Figure 1: Levels of disease severity, tissue remodeling, angiogenesis and endothelium stability markers in metastatic lungs. Mice from 4T1 and 4T1+ASA/Cl groups were euthanized in the terminal 5th week of the disease and their primary tumours and lungs were excised and homogenized. After determination of protein concentration, primary tumours and lungs homogenates from each experimental group were pooled together in such a way that an equal amount of protein was taken in equal volume from each animal in the particular group (n=6) to assure equal representation of each mouse in the pooled sample. Subsequently, an equal amount of protein from control 4T1 and ASA-Cl group was subjected to SDS-PAGE in the reducing conditions. Then, membranes were blocked with 5% milk and incubated with appropriate primary and secondary antibodies directed against markers of disease severity (PGI_2S (Prostacyclin Synthase), TGFβ1 (Transforming Growth Factor β1), (COX-2) (Cyclooxygenase-2)), the tissue damage/remodeling (vWF (von Willebrand Factor) and Metalloproteinases 2, 9 and 14 (MMP-2, MMP-9, MMP-14), markers of angiogenesis (VEGFA (Vascular Endothelial Growth Factor A), EphA1 (Ephrin A1), Angiopoetins 1 and 2 (Ang-1 and Ang-2), CD31 (cluster of differentiation 31), eNOS (endothelial Nitric Oxide Synthase), VE-CAD (Vascular Endothelial Cadherin), and markers of endothelium stability (Slit2 (Slit homolog 2) and its endothelium-specific receptor ROBO4 (Roundabout homolog 4) that interacts with N-terminal but not C-terminal Slit2). The images present the fold change of each protein of interest in 4T1+ASA/Cl group vs control 4T1 group. Equal protein loading was controlled after electrophoresis, and transfer for all gels and membranes using a stain free-technique provided by Bio-Rad, as described in *Materials and Methods*.

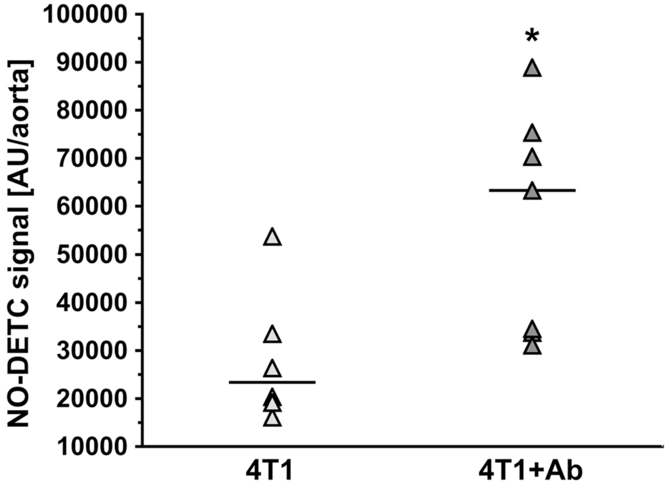

Supplementary Figure 2: NO production by the aorta after intravenous injection of 4T1 breast cancer cells. Mice received saline (control 4T1; n=6) or were injected with platelet-depleting antibody (4T1+Ab, n=7) 15 min before i.v. injection of 4T1 breast cancer cells. After euthanization in the 13th day after 4T1 cell inoculation, the aortas were excised, cleaned from the surrounding tissue and *ex vivo* NO production was measured by NO spin trapping and subsequent detection with EPR. The spectra were obtained using an X-band EPR spectrometer (EMX Plus, Bruker, Germany), equipped with a rectangular resonator cavity H102. Instrument settings were: centre-field (B_0) 3276G, sweep 115G, microwave power 10 mW, modulation frequency 100 kHz, amplitude modulation 8 G, sweep time 60 s, and number of scans 4. Signals were quantified by measuring the total amplitude of the NO-DETC after correction of baseline. The data were analysed with D'Agostino and Pearson omnibus normality test and F test for equal variances and based on their results the means in control 4T1 and 4T1+Ab mice were compared with two-tailed Mann-Whitney test (28193±13945 AU for 4T1 mice, n=6 and 56737±23412 AU for 4T1+Ab mice, n=7, P=0.014). Data are presented as means ± SD.
